# Supplementary material for: Behavior of Muscle-Derived Stem Cells on Silica Nanostructured Substrates
Source: Nanomaterials (Basel). 2020 Aug 22;10(9):1651. doi: 10.3390/nano10091651 (PMC7558836; doi:10.3390/nano10091651)
Supplement: Supplementary file 1 [file nanomaterials-10-01651-s001.pdf]

nanomaterials

Supporting Information

Behavior of Muscle-derived Stem Cells on Silica Nanostructured Substrates

Hyo-Sop Kim, Bit Na Lee, Sangdun Choi, Moon Suk Kim*, Jae-Ho Kim*

Department of Molecular Science and Technology, Ajou University, Suwon 16499, Korea, ikari06@ajou.ac.kr (H.S.K.); newri21@naver.com (B.N.L.); sangdunchoi@ajou.ac.kr (S.C.)

* Correspondence: moonskim@ajou.ac.kr; Tel.: +82-(31)-219-2608; Fax: +82-(31)-219-3931; jhkim@ajou.ac.kr; Tel.: +82-(31)-219-2517; Fax: +82-(31)-219-2516

Hyo-Sop Kim and Bit Na Lee are equal first authors.

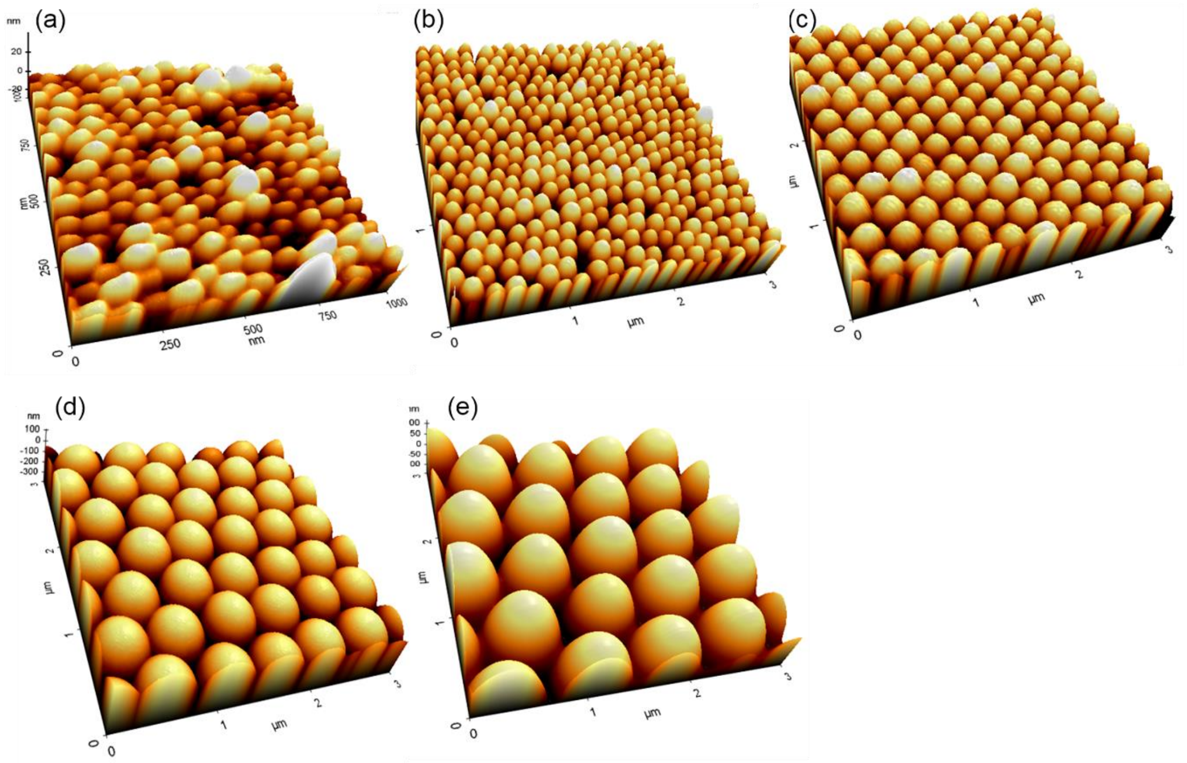

Figure S1. AFM images of (a) SN-60, (b) SN-150, (c) SN-300, (d) SN-500, and (e) SN-700. Scale indicated (a) 1 μm and (b-e) 3 μm.

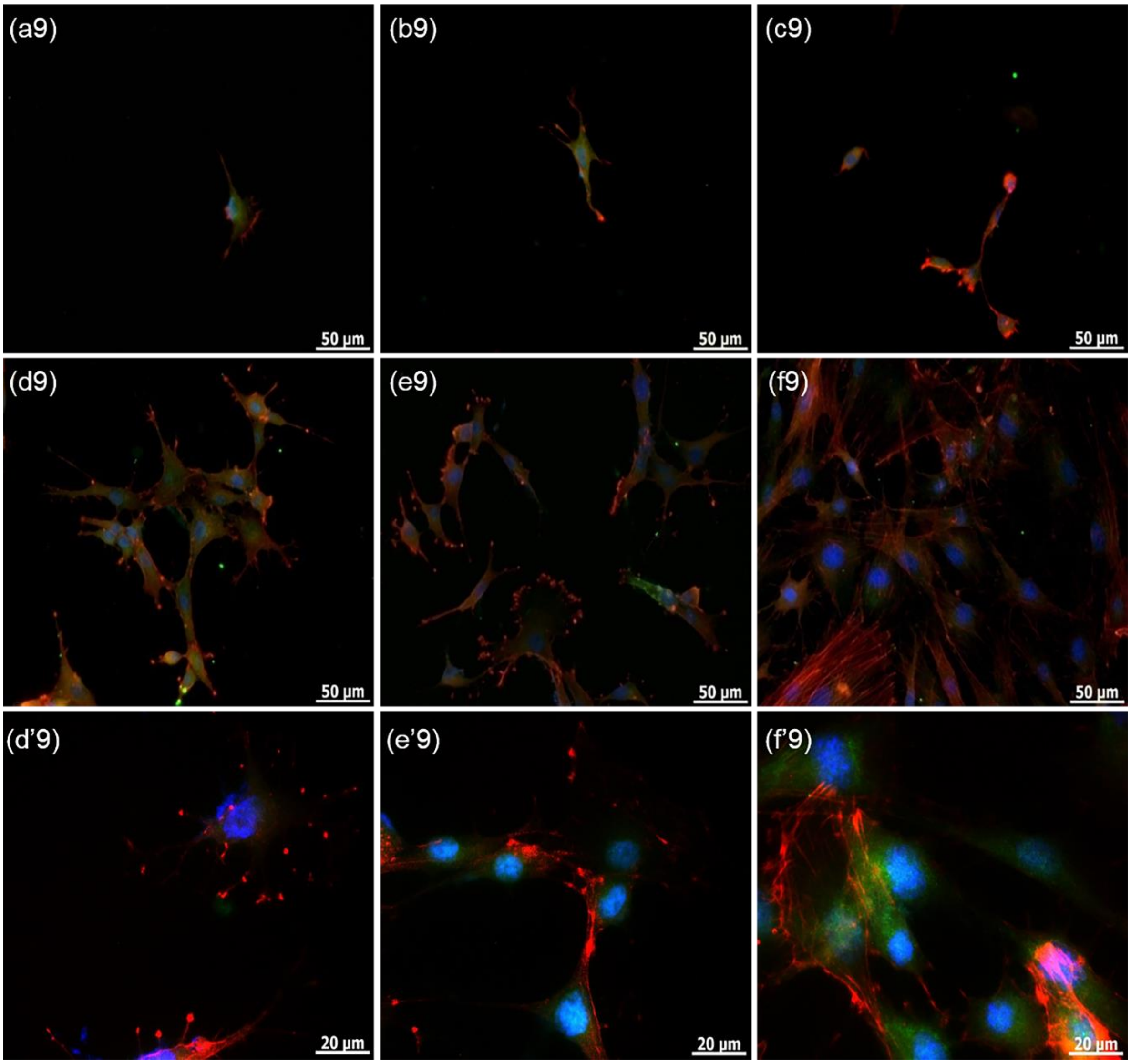

Figure S2. Actin-focal adhesion immunostaining in rMDSCs grown on SN at 9 d. (a) SN-60, (b) SN-150, (c) SN-300, (d) SN-500, (e) SN-700, and (f) glass. DAPI (blue), actin (red); FAC (vinculin; green). Magnification, x400; scale bar = 50 µm. (d'9), (e'9), and (f'9) are enlarged images (magnification, ×1000).

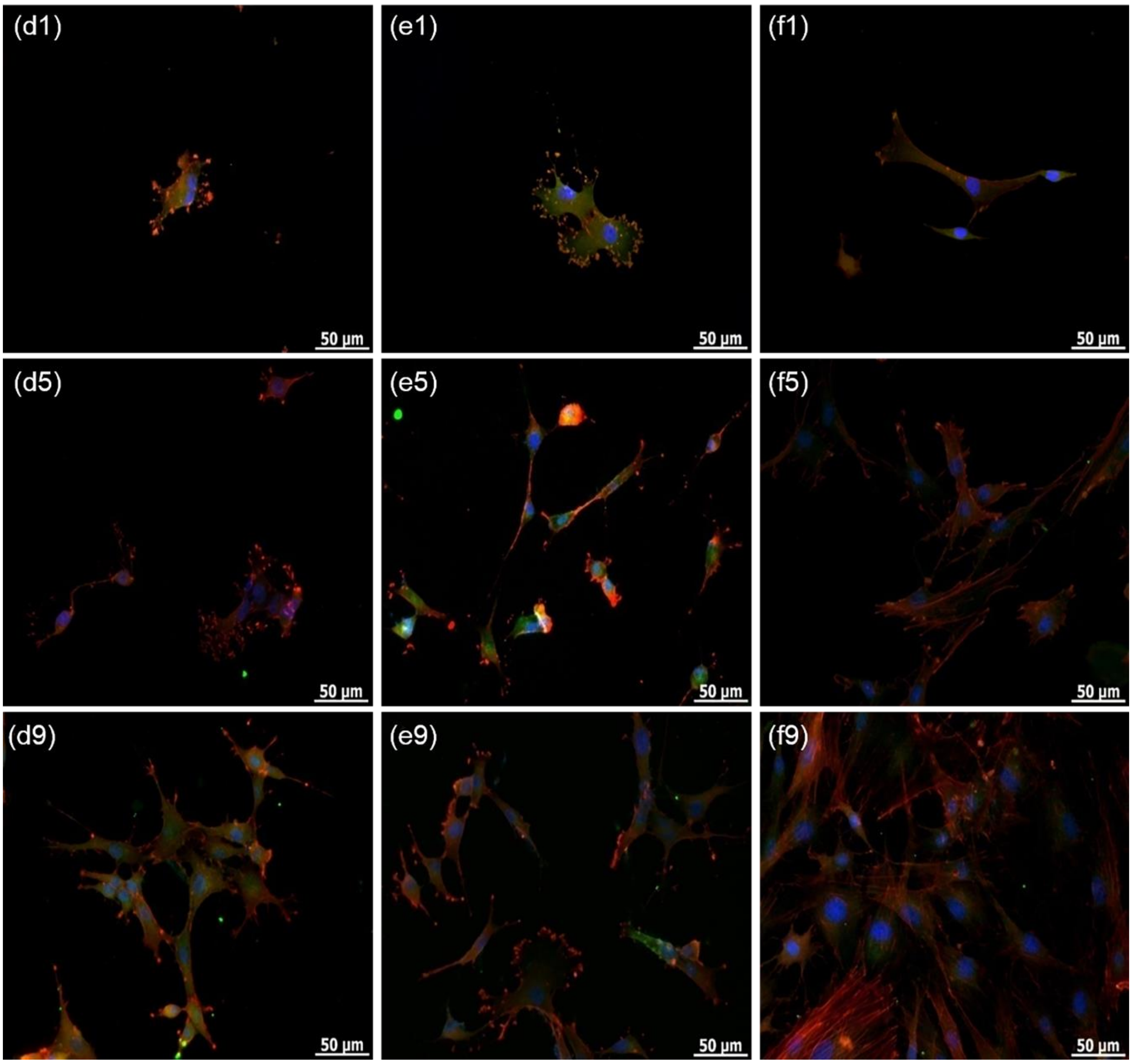

Figure S3. Actin-focal adhesion immunostaining in rMDSCs grown on SN for 1, 5, and 9 d. (d) SN-500, (e) SN-700, and (f) glass. The numbers indicate 1, 5, and 9 d. DAPI (blue), actin (red); FAC (vinculin; green). Magnification, x400; scale bar = 50 μm.

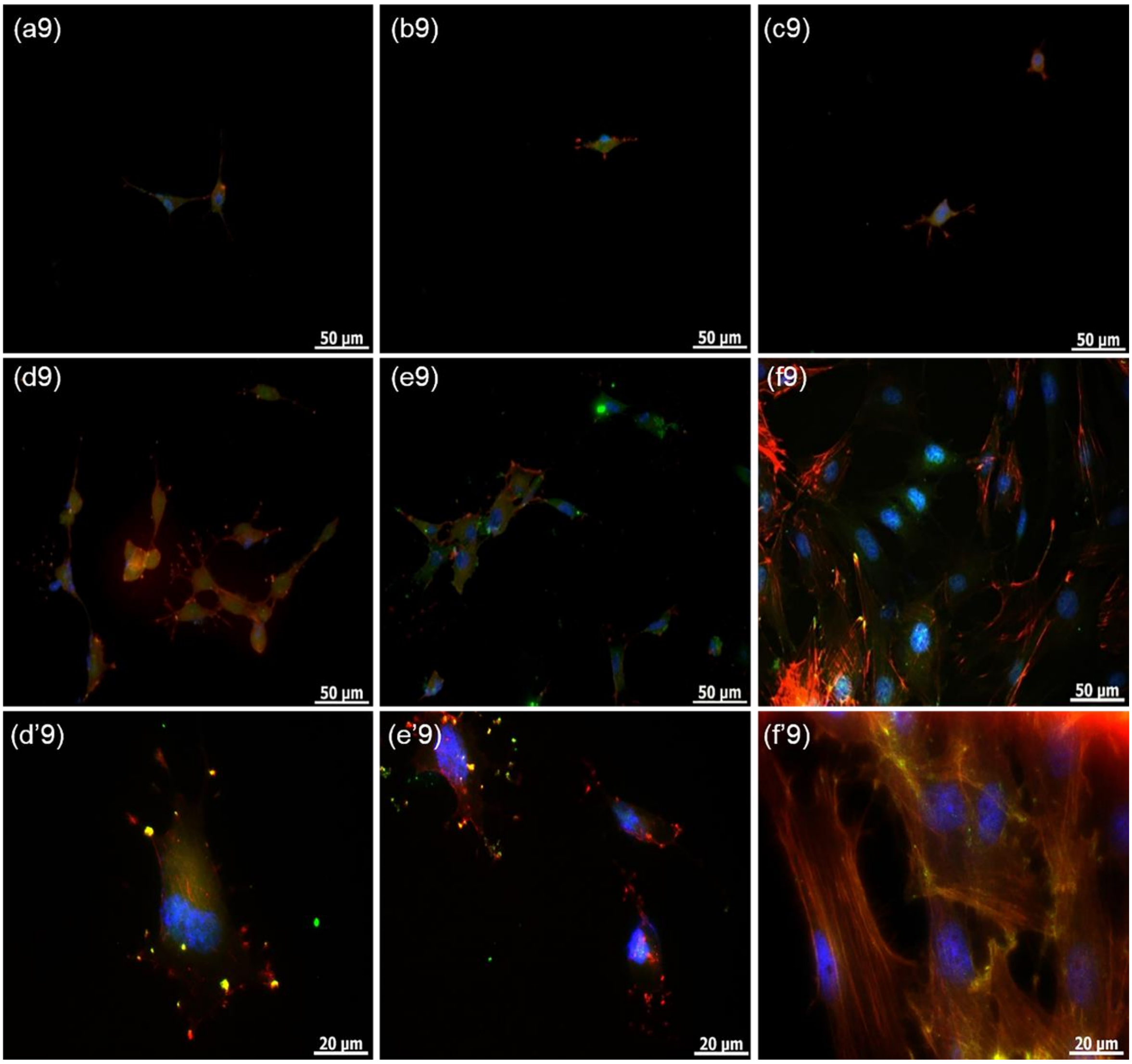

Figure S4. Actin-integrin β1 immunostaining in rMDSCs grown on SN at 9 d. (a) SN-60, (b) SN-150, (c) SN-300, (d) SN-500, (e) SN-700, and (f) glass. Blue, DAPI; red, actin; green, integrin β1. Magnification, ×400; scale bar = 50 μm. (d'9), (e'9), and (f'9) are enlarged images (magnification, ×1000).

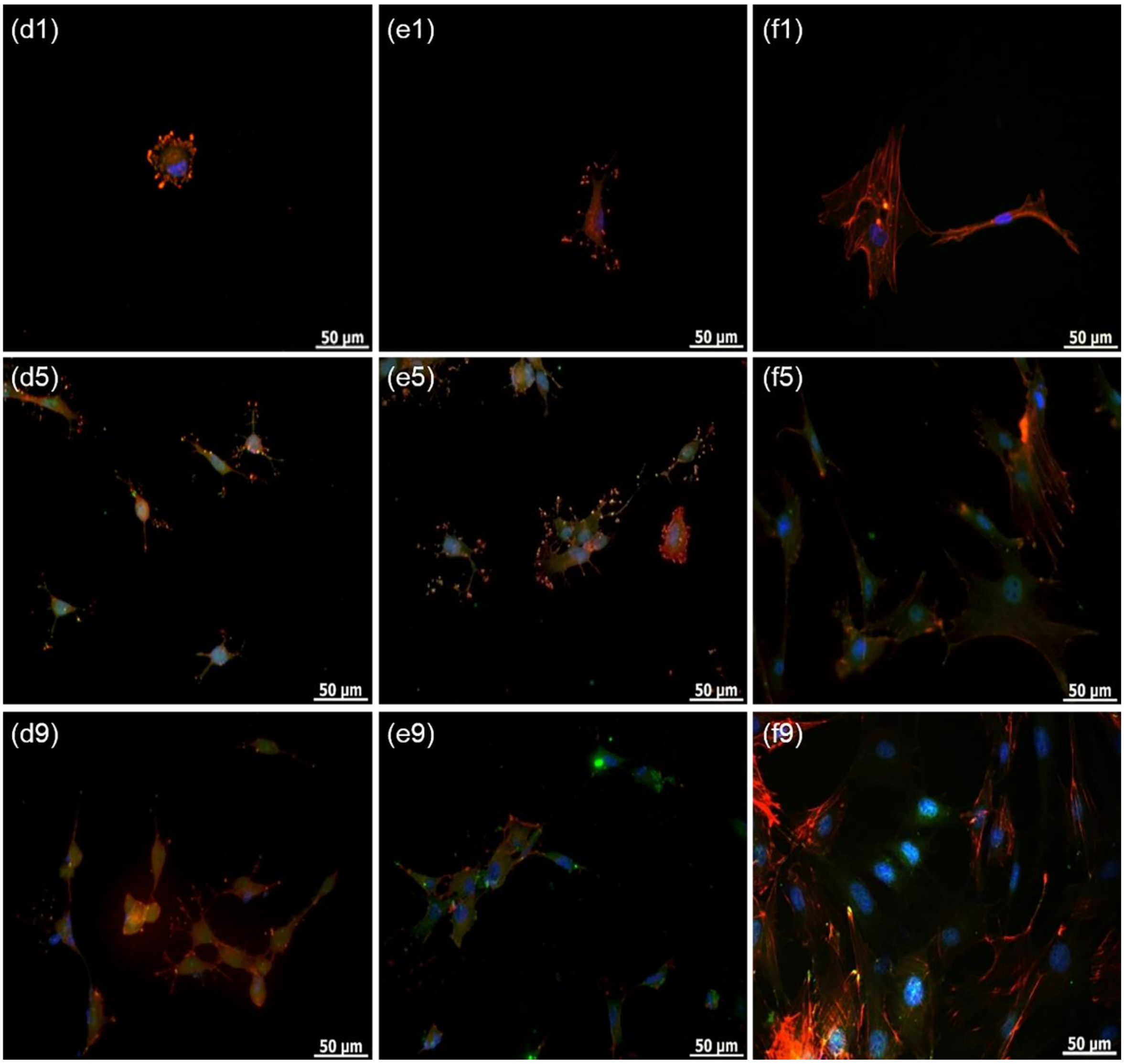

Figure S5. Actin-integrin β1 immunostaining in rMDSCs grown on SN for 1, 5, and 9 d. (d) SN-500, (e) SN-700, and (f) glass. The numbers indicate 1, 5, and 9 d. Blue, DAPI; red, actin; green, integrin β1. Magnification, ×400; scale bar = 50 µm.
